# Metachronous Bladder Metastases From Renal Cell Carcinoma: A Case Report

**DOI:** 10.7759/cureus.110177

**Published:** 2026-06-03

**Authors:** Oualid Bounouar, Anouar El Moudane, Ahmed Jdaini, Atif Lifa, Ali Barki

**Affiliations:** 1 Urology, Centre Hospitalier Universitaire Mohammed VI Oujda, Oujda, MAR; 2 Urology, Mohammed VI University Hospital, Oujda, MAR; 3 Research, Faculté de Médecine et de Pharmacie d'Oujda, Oujda, MAR

**Keywords:** case report, gross hematuria, metachronous bladder metastases, open nephrectomy, renal cell carcinoma

## Abstract

Renal cell carcinoma (RCC) is a common tumor of the urinary system, with several pathological subtypes, including clear cell, papillary, and chromophobe RCC. While RCC has the potential to metastasize to nearly any organ, metastasis to the urinary bladder is rare. We describe the case of a 76-year-old man who underwent right radical nephrectomy in July 2023. A follow-up CT scan was performed in October 2023, which showed no evidence of metastasis, but the patient was subsequently lost to follow-up. Twenty-five months later, he presented to the emergency department with hematuria. Imaging revealed a mass at the bladder neck and posteroinferior wall, along with additional metastatic lesions in the lungs, liver, and right adrenal gland. Transurethral resection was performed, and histopathology confirmed metastatic clear cell RCC. This case highlights the need to consider bladder metastasis in patients with a history of RCC who present with bladder lesions.

## Introduction

Kidney cancer ranks as the 14th most frequent cancer globally. Its occurrence varies widely, being more common in men, in individuals over 75 years of age, and in Western regions such as Europe and North America [1].

In France, an estimated 17,141 new cases of kidney cancer were reported in 2023, representing a 199% increase in incidence between 1990 and 2023. This increase was particularly marked among individuals under 50 years of age and among men, reflected by a male-to-female incidence ratio of approximately 2:1 [2,3]. RCC is widely recognized for its ability to metastasize and its tendency to spread to uncommon locations.

From a histological perspective, the vast majority (90%) of kidney cancer cases are RCC. Among these, the main subtypes are clear cell RCC (70%), papillary RCC (10-15%), and chromophobe RCC (5%). Other subtypes are rare, each accounting for 1% or less of all cases [4].

The most frequent sites for renal cell carcinoma (RCC) metastasis include the lungs, bones, liver, lymph nodes, and brain [5]. In comparison, metastasis to the urinary bladder is exceptionally rare. When present, bladder metastases may occur synchronously with the primary tumor or develop metachronously, and they can manifest as isolated lesions or as part of multifocal metastatic disease.

Pathologically, bladder lesions from RCC must be distinguished from primary urothelial carcinoma and from invasion of the bladder by adjacent tumors. Key distinguishing features include clear-cell morphology, evidence of vascular invasion, and an immunoprofile consistent with renal origin.

## Case presentation

A 76-year-old man with no notable medical history was admitted to the emergency department of our hospital during the last week of June 2023 due to macroscopic hematuria. The patient was hemodynamically unstable. Initial vital signs were as follows: temperature 37°C; heart rate 115 bpm; and blood pressure 100/50 mmHg. On physical examination, there were no other signs of bleeding besides hematuria.

A blood test showed normal kidney function (creatinine of 1 mg/dL) with anemia (hemoglobin 9 g/dL). Urine analysis showed a markedly elevated red blood cell count and an inflammatory syndrome with a CRP of 89 mg/L, while the white blood cell count was normal (Table 1).

**Table 1:** Baseline hematologic and biochemical parameters of the patient.

Test	Result	Normal Range
Hemoglobin	9 g/dL	13-17 g/dL
Hematocrit	40%	38-50%
White blood cells (WBCs)	9 × 10³/µL	4-11 × 10³/µL
Platelets	220 × 10³/µL	150-400 × 10³/µL
Creatinine	1 mg/dL	0.7-1.3 mg/dL
Blood urea nitrogen (BUN)	18 mg/dL	7-20 mg/dL
Sodium	138 mmol/L	135-145 mmol/L
Potassium	4.1 mmol/L	3.5-5.0 mmol/L
C-reactive protein (CRP)	89 mg/L	<10 mg/L

The contrast computer tomography (CT) scan identified a 4.2 × 2.9 cm right renal tumor located in the lower pole of the kidney; the lesion exhibited extension into both the renal artery and renal vein, consistent with vascular invasion. No evidence of regional lymphadenopathy or distant metastases was identified (Figure 1). The tumor was classified as pT3aN0M0 according to the tumor, node, metastasis (TNM) classification (Union for International Cancer Control (UICC)) [6], and the radiographic appearance raises the possibility of a clear cell renal carcinoma.

**Figure 1:**
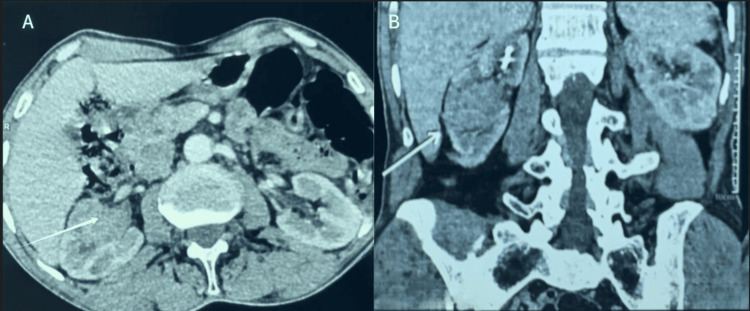
CT scan reveals a right renal mass classified as pathological stage T3a according to the TNM classification system of the UICC. (A) Transverse CT scan showing the right renal tumor before the nephrectomy. (B) Sagittal CT scan showing the renal tumor. TNM staging system for kidney cancer, adapted from Reference [6]. TNM: tumor, node, metastasis; UICC: Union for International Cancer Control

The patient underwent a right-sided nephrectomy using an open retroperitoneal technique.

Pathological examination revealed a well-circumscribed, solid, elastic mass measuring 5 cm, with coloration ranging from white to orange. The tumor was classified as Grade 3 according to the International Society of Urological Pathology (ISUP) grading system [7], with evidence of a microthrombus in the renal vein and no sign of arterial invasion. Immunohistochemically, the tumor cells were positive for CD10 and PAX8 and negative for CK7, CD117, and CK20 (Figure 2). The patient underwent the follow-up protocol of a chest and abdomen CT scan at three months with no sign of recurrence or metastasis. Unfortunately, the patient was subsequently lost to follow-up.

**Figure 2:**
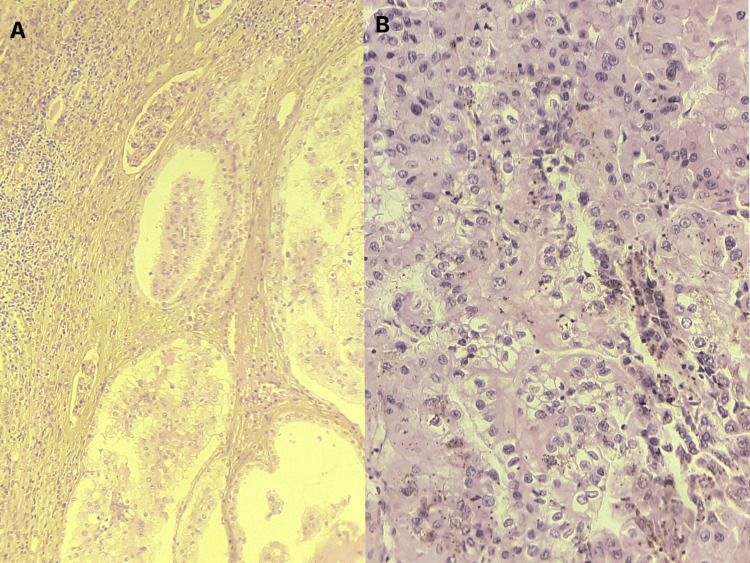
Histological sections of the nephrectomy specimen and the bladder lesion. (A) Renal tumor was classified as Grade 3 according to the ISUP grading system. (B) Bladder lesion demonstrates a similar proliferation of clear and eosinophilic cells. ISUP classification, adapted from Reference [7]. ISUP: International Society of Urological Pathology

He presented to the emergency department 25 months later, with another episode of hematuria. CT of chest, abdomen, and pelvis revealed a mass on the bladder neck and posteroinferior bladder wall measuring 5.2 × 4.7 cm with additional metastatic sites in the lung, liver, and right adrenal gland (Figure 3).

**Figure 3:**
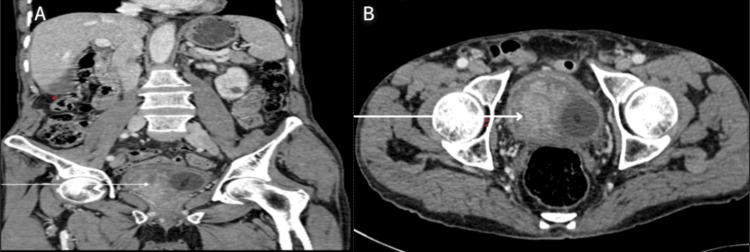
CT scan reveals a mass in the bladder. (A) Sagittal CT scan showing the bladder metastases. (B) Transverse CT scan showing the bladder metastases.

The patient underwent en bloc resection of the bladder tumor. Histopathology showed vascular emboli and tumor necrosis, with no perineural invasion and no detrusor involvement. Immunohistochemically, the tumor cells were positive for PAX8 and CD10 and negative for CK7, CD117, GATA3, and CK20 (Figure 2). Following surgery, pembrolizumab was recommended by the oncology team at our institution in a multidisciplinary consultation meeting. Unfortunately, the patient and his family refused any form of treatment.

## Discussion

RCC most commonly spreads to the lungs, occurring in over half of cases. Other frequent sites of metastasis include the lymph nodes, liver, and bones, which are involved in roughly 20-30% of patients. Less commonly, the disease can spread to the brain (around 10%) and, in some cases, to endocrine organs such as the thyroid and pancreas, while the bladder only accounts for approximately 2% of RCC metastases [8,9]. In 2015, a total of 65 cases of bladder metastases had been documented. A retrospective analysis of these 65 cases of RCC metastasizing to the bladder showed that these intravesical metastases may be synchronous or metachronous, occurring, respectively, in 23% and 77% of reported cases. Among metachronous cases, the median interval between the initial RCC diagnosis and the development of bladder metastasis was 33 months. Pathological evaluation indicated that clear cell carcinoma was the predominant histological subtype, accounting for 92% of cases, and all bladder tumors were confirmed to be metastases originating from RCC, with approximately two-thirds of cases presenting as superficial (non-muscle-invasive) disease, and 62% present as a solitary lesion, whereas 38% occur as part of disseminated metastatic disease [10].

The precise mechanism behind bladder metastasis from RCC remains unclear, but four main pathways have been proposed. The first is “drop metastasis,” in which tumor cells travel down the urinary tract and implant directly into the bladder. This is supported by cases showing bladder metastases near the ureteral orifice after nephrectomy, or polypoid RCC growth disrupting the urothelium, allowing tumor cells to float and implant [11]. Other potential routes include direct hematogenous spread, lymphatic dissemination, and retrograde venous spread, where tumor emboli from the renal vein travel through its venous connections [12]. In our patient, multiple metastatic sites, including the bladder, lung, liver, and right adrenal gland, suggest that hematogenous spread likely accounts for the systemic metastases, while drop metastasis explains the bladder involvement. Given the overlap between these pathways, it is possible that more than one mechanism contributed to the unique pattern of metastasis observed.

Having other metastatic lesions at the time bladder metastasis is detected, or developing bladder metastasis within a year after initial treatment of RCC, are both recognized as independent factors influencing prognosis [10].

The lack of conclusive evidence has resulted in the absence of established guidelines for treating bladder metastasis from RCC. Treatment of RCC metastases is individualized for each patient. In cases of bladder metastasis, the recommended approach is typically close monitoring after transurethral resection or partial cystectomy. Radical cystectomy may be considered, but it is generally reserved for lesions confirmed to invade the bladder muscle. Furthermore, if metastases are present in other organs at the time bladder involvement is detected, systemic therapies, such as immunotherapy, are indicated in addition to local treatment.

## Conclusions

Metachronous metastasis of RCC to the bladder is an exceptionally rare occurrence that clinicians should be aware of. Diagnostic confirmation in such rare presentations can be challenging and requires careful pathological evaluation. Correlation of morphology with a comprehensive immunohistochemical panel. The exact pathway by which RCC spreads to the bladder remains uncertain. Treatment strategies are still debated, but patients with a solitary bladder lesion arising metachronously generally have a favorable prognosis, whereas those with synchronous or multiple metastases tend to have a poorer outcome.
